# Genomic Evaluation of AML—Main Techniques and Novel Approaches

**DOI:** 10.3390/jcm14165685

**Published:** 2025-08-11

**Authors:** Dinnar Yahya, Milena Stoyanova, Mari Hachmeriyan, Mariya Levkova

**Affiliations:** 1 Department of Medical Genetics, Faculty of Medicine, Medical University of Varna, 9000 Varna, Bulgaria; 2 Laboratory of Medical Genetics, UMHAT “Sv. Marina”, 9000 Varna, Bulgaria

**Keywords:** acute myeloid leukemia, genetic markers, next-generation sequencing, artificial intelligence, machine learning

## Abstract

The genetic diversity of acute myeloid leukemia creates a major obstacle for current research and clinical practice. Despite advances in molecular genetic techniques, various omics approaches, and artificial intelligence, developing a universal algorithm to thoroughly assess each clinical case remains difficult. Starting with current recommendations and classifications, this narrative review highlights the most important diagnostic options available today, new opportunities that are emerging, and the challenges in diagnosing and managing this complex disease.

## 1. Introduction

Acute myeloid leukemia (AML) encompasses a range of diseases characterized by the abnormal growth of progenitor cells in the myeloid lineage. According to the defined mechanisms of etiology and pathogenesis, it is recognized that, at the cellular level, this condition is a single-core genetic disorder linked to various genetic mutations and epigenetic changes. These primarily acquired changes often result in hematopoiesis disorders related to proliferation, differentiation, maturation, cell cycle regulation, and apoptosis. AML is distinguished by notable genetic diversity, an aggressive clinical progression, and unpredictable outcomes [1].

### 1.1. Classifications

Two widely used classifications—the World Health Organization (WHO) and the International Consensus Classification (ICC)—should be considered for a comprehensive understanding of the genetic nature of AML. WHO was the first classification to incorporate genetic information into the characterization of the different subtypes of AML, in 2001 [2]. Due to its comprehensiveness and evolution with several revisions over time, it is considered the gold standard. The latest and currently valid revision is the fifth [3], announced in 2022 (Table 1).

Genetic alterations are crucial in the WHO classification, organizing cases into distinct subtypes. Unlike earlier systems, the WHO does not stipulate a minimum number of blasts to define genetic abnormalities. This highlights the importance of driver mutations, irrespective of clone size, during evaluation. Specific genetic events correlating with morphological findings are sufficient for diagnosing AML, regardless of myeloblast counts. The subgroup AML with unique genetic alterations is also introduced, which may develop into a separate subtype in future classifications. These factors underscore the importance of chromosomal or molecular alterations, as well as clinical and morphological characteristics, in diagnosis and treatment strategies [3].

In 2022, the latest edition of the ICC was published [4]. It demonstrates remarkable similarity with the WHO classification, with some differences regarding minimum blast requirement, and additional single-gene and chromosomal characteristics [5].

Also of interest is the “softening” of the boundaries between myelodysplastic syndrome (MDS) and AML in both classifications, including the presence of the so-called MDS/AML form in ICC. This perspective on the two conditions helps us to understand the potential transition from MDS to AML and the appropriate course of action in these cases.

### 1.2. Risk Stratification

Given the heterogeneous, complex, and case-by-case nature of AML, developing algorithms to clarify cases and streamline diagnostic and therapeutic processes is crucial. Alongside clinical, laboratory, and statistical assessments, routine practice utilizes ELN risk stratification (Table 2). Accepted as a gold standard, it predicts risk levels: favorable, intermediate, or unfavorable, with overall 5-year survival rates of 60%, 40%, and 20%, respectively [6]. The genetic findings confirm the importance of chromosomal and single-gene mutations in the 2022 WHO classification. Strikingly, the most common genetic changes indicate unfavorable risk. This trend has persisted in recent revisions. Some authors believe this stratification method is inadequate, especially for patients over 55, who are a significant portion of the affected population [7]. This indicates a need for enrichment with additional genetic and cytogenetic markers beyond the framework of this updated stratification, which will likely be incorporated into its next version.

### 1.3. Recommendations and Guidelines

Cytogenetic evaluation of the genetic background of an AML case undeniably contributes to outcome prognosis and therapeutic choice [8]. With chromosomal markers in the WHO classification and ELN risk stratification, conventional cytogenetic analysis (CCA) is still essential. The method provides a whole-genome assessment of large-scale alterations at a resolution of 5–10 megabase pairs, including deletions, duplications, inversions, balanced and Robertsonian translocations, ring, iso-, and marker chromosomes. It can evaluate clones with 10% sensitivity, depending on the number of metaphase plates analyzed [9]. The usually recommended turnaround time (TAT) is 7–21 days [10]. Regarding its price, it varies, but can be as low as USD150 [11]. However, the presence of molecular genetic markers indicates the need for higher-resolution methods. Two genomic approaches have proven useful for discovering and understanding molecular genetic markers: SNP (single-nucleotide polymorphism) microarray and next-generation sequencing (NGS) [12,13,14,15,16]. ELN 2022 recommends screening for over ten molecular genetic markers in all newly diagnosed AML patients, including *FLT3*, *NPM1*, *CEBPA*, *RUNX1*, *TP53*, and others, and several gene fusions (*PML::RARA*, *CBFB::MYH11*, *RUNX1::RUNX1T1*, *BCR::ABL1*, and *KMT2A* rearrangements), in addition to CCA. Twenty more genes fall within these recommendations to be tested at diagnosis [6,16]. This need for extensive genetic information drives the demand for a comprehensive molecular analysis or a combination that meets all requirements.

Several approaches to genetic assessment are currently used in conjunction with CCA. These methods aim to identify the subtle single-gene variations that drive the leukemic process and its evolution. One such method is NGS, which currently concentrates on gene panels featuring well-known markers for AML.

## 2. Gene Panels

NGS is, unequivocally, the most commonly used method for molecular genetic assessment of AML patients. It is now a routine diagnostic tool with many pre-selected gene panels available on the market. Panel testing enables the simultaneous evaluation of multiple disease-associated genes, thereby reducing the cost of the current standard of care. In addition to this, it contributes to the selection of target therapy, response assessment, and evaluation of the risk of relapse after allogeneic stem cell transplantation [17]. Aside from single-nucleotide variants (SNVs), NGS can also detect copy number alterations (CNAs), translocations, indels, and even loss of heterozygosity in its latest updates [18,19,20]. The tendency to improve and update its analytical scope is promising for selecting a suitable molecular assay for AML patient assessment.

Current recommendations support the use of NGS as a molecular genetic method for classification and risk stratification, in addition to CCA [6]. The currently available gene panels differ in the number of genes included, with the Qiagen Human Myeloid Neoplasms Panel and Illumina AmpliSeq Myeloid Panel being among the most comprehensive ones [21]. However, Genomic Medicine Sweden recently introduced an even larger panel that showed high detection sensitivity of all known clinically relevant genes and novel ones, even overcoming the difficult GC-rich regions [22]. This promotes NGS forsuccessful incorporation in routine clinical assessment, especially with the ability to provide rapid results for 2–7 days [23,24]. Additionally, it is also frequently the choice in the work-up of pediatric cases [25,26]. Still, the relatively high price should be taken into account, varying from a few hundred to a few thousand USD per sample [27,28]. As for validation, the high concordance deems post-NGS Sanger sequencing unnecessary, which reduces costs and technical difficulty [29]. Another approach suggested for this aim is also through machine learning models, which can assess the false-positive result rate [30].

Several studies also demonstrate the ability of this method to detect measurable residual disease (MRD), which holds both predictive and prognostic value in AML [31]. The variant allele frequency (VAF) reported in these studies reaches as low as 0.01%, which is essential for monitoring treatment response and the risk of relapse [32,33,34,35,36,37,38,39]. Other standard MRD-detecting approaches have been implemented for a long time, such as multiparallel flow cytometry and digital or quantitative polymerase chain reaction [40]. However, a deeper investigation of its advantages and limitations is necessary before NGS can potentially replace these methods in the future as a first-line management and monitoring algorithm approach [41,42]. Currently, it is included as an exploratory method with defined technical requirements, such as error-correcting approaches, in the ELN 2022 guidelines [6].

## 3. Whole-Exome Sequencing

Another possible implementation of NGS is to assess the entire coding sequence in the genome through whole-exome sequencing (WES), which has been a cornerstone in advancing the molecular understanding of AML. By focusing on the protein-coding regions of the genome, WES proved to be a cost-effective research tool that successfully identified a wide array of novel and recurrent driver mutations, particularly in genes responsible for epigenetic regulation, splicing, and the cohesin complex. This was especially transformative for cytogenetically normal AML, moving the field beyond a purely structural view of the genome and helping to establish the molecular–mutational foundation for current risk stratification models [43,44,45]. In clinical practice, WES remains valuable for discovering new variants, and as a diagnostic tool when targeted gene panels yield inconclusive results. However, its utility is constrained by significant limitations. WES is inherently limited in detecting large structural variants (SVs) and CNAs, which are crucial for AML diagnosis and prognosis. Furthermore, it is blind to 99% of the genome, which comprises non-coding regions that may contain disease-relevant regulatory elements. With 5–10% VAF, it lacks the high sensitivity required for MRD monitoring [46]. Pricewise, it can cost up to USD 2000 [47]. Also, the usual TAT is a few weeks, which might be too long for AML cases [48].

## 4. Whole-Genome Sequencing

Whole-genome sequencing (WGS) addresses genetic information as a whole, including both coding and non-coding regions. It represents the next step toward a comprehensive, unified genetic assessment of AML, capable of detecting all classes of genetic alterations in a single assay [49,50,51]. Landmark studies have demonstrated its diagnostic superiority over conventional methods; WGS shows high concordance for all established risk-defining abnormalities, while also uncovering additional, clinically relevant genetic information in up to 25% of patients [49]. This increased diagnostic yield frequently leads to more accurate risk re-stratification, which can directly alter patient management. Despite this promise, significant hurdles hinder its routine clinical implementation. The primary challenge has shifted from variant detection to interpretation, particularly for the vast number of variants of unknown significance (VUS) discovered in non-coding regions. Understanding their functional impact requires sophisticated bioinformatics and the integration of multi-omics data [52]. Furthermore, there are practical barriers, such as high costs—up to USD 4000, and the TAT, which can be a few days [49] but usually takes even longer than WES. Regarding MRD monitoring, the VAF of 5–10% is a current limitation similar to WES. In addition, massive data storage needs and the need for standardized regulatory and reporting frameworks must be overcome before WGS can be widely adopted as the new standard of care [47,53,54].

## 5. SNP-Array

SNP microarray is a genomic method that uses probes for SNPs to detect genomic changes. It goes beyond traditional chromosomal microarray analysis by detecting unbalanced quantitative changes such as microdeletions and amplifications, including loss of heterozygosity and ploidy [14,15]. SNP microarray also identifies interstitial deletions found in about 25% of common fusion genes. However, these fusions are balanced changes, and cannot be detected without accompanying deletions. Loss of heterozygosity, where a normal allele is replaced by duplication of the mutant allele, along with abnormalities such as hypodiploidy and hyperdiploidy, is crucial in leukemic pathogenesis, impacting treatment decisions. Another advantage over CCA is that it provides insights into ambiguous findings, such as marker and ring chromosomes. However, it cannot report balanced changes, and may miss clones with less than 10% cell representation, making it unsuitable for MRD monitoring. Also, while academic studies reached a cost of USD 40 per sample [55], diagnostic use in a clinical setting showed a higher price, of several hundred USD, with a TAT of two to three weeks. Additionally, the 5–10 kilobases resolution limit prevents the detection of minor key AML mutations, such as *FLT3*, *NPM1*, and *C-KIT*, which affect one, or a few, base pairs (bp). Thus, SNP microarray is insufficient as a sole molecular genetic method, indicating a need for supplementary methods [15].

## 6. Optical Genome Mapping

Still, the combination of CCA and targeted NGS fails to recognize certain events, particularly due to the poor resolution achieved through the former method. A potential solution to this problem could be the relatively new optical genome mapping (OGM). It is not based on sequencing, but on recognizing fluorescently labeled DNA motifs. The VAF is 5%, and the resolution is 500 bp. The combination with targeted NGS, as demonstrated in a study by Yang et al., resulted in a double number of findings compared to CCA, regarding CNAs and SVs [56]. Other studies have shown a similar potential for the method, including risk re-stratification and altering clinical management [57,58,59]. Of course, given the VAF, it would not be applicable for MRD monitoring, but the method could establish itself as a new gold standard for cytogenetic changes. This is especially valid, given the lack of need for cell cultivation and a TAT of 7–14 days [60]. Its only limitation compared to CCA is the reduced sensitivity for Robertsonian translocations. Perhaps the main reason for its secondary-line position in routine genetic assessment is the higher cost, approximately USD 500 per sample [58]. This could present a limitation for countries with developing economies and smaller centers. Nevertheless, a framework has recently been established for the centers that transition to OGM instead of conventional methods—CCA, fluorescent in situ hybridization, and microchip array—to establish quality control standards and facilitate implementation [61].

## 7. Long-Read Sequencing

Long-read sequencing, also known as third-generation sequencing, is characterized by its ability to analyze much longer DNA fragments compared to the widely used short-read technologies. This provides long-read sequencing with a distinct advantage in detecting large SVs, which are common in hematologic malignancies but are often difficult to identify using short-read sequencing [62]. Currently, two platforms dominate the long-read sequencing market: Pacific Biosciences’ single-molecule real-time sequencing and Oxford Nanopore Technologies’ nanopore sequencing [62]. Although long-read sequencing offers substantial benefits, it is not yet widely applied in the molecular genetic testing of hematologic malignancies. However, Wang et al. applied RNA nanopore sequencing to differentiate leukemia subtypes in children, achieving 96.2% accuracy for AML subtypes and 94.1% accuracy for major B-lineage acute lymphoblastic leukemia subtypes [63]. Additionally, nanopore sequencing has been successfully used to detect SVs in AML patients. For example, it enabled the detection of a novel inv(16) in one patient, and a translocation between chromosomes 10 and 12—a variant that was detected by CCA but missed by routine short-read NGS, in another [64,65].

However, the technique has certain limitations. Historically, it has had higher raw read error rates compared to short-read sequencing. While recent improvements, such as duplex sequencing and advanced base-calling algorithms, have significantly enhanced accuracy, validation of results obtained by long-read sequencing remains a widely accepted practice, even though it is not formally mandated [66,67]. Also, the method is still challenged by lower-level clones with a VAF of ≥10% [68]. Another limitation for its broader implementation is the variable yet higher per-sample costs, lower throughput, and the need for specialized bioinformatics tools and expertise [62,67]. Nevertheless, long-read sequencing holds great promise for advancing molecular diagnostics in hematologic malignancies, and is expected to be widely adopted soon.

## 8. Hi-C Analysis

High-throughput chromosome conformation capture (Hi-C) analysis is emerging as a key role in AML by enabling the genome-wide detection of chromatin structural aberrations—large-scale rearrangements, gene fusions, and compartment shifts that may be missed by CCA or short-read sequencing methods [69,70,71]. Across institutional series, this technique showed complete concordance with ELN-defining translocations identified by standard workflows, and uncovered additional clinically actionable fusions in 15–40% of cases with cryptic or complex karyotypes, underscoring its value as a diagnostic adjunct [69,72]. Beyond classical rearrangements, Hi-C interrogates higher-order chromatin features, such as topologically associating domains (TADs) and enhancer–promoter loops. Pathological TAD disruption and enhancer hijacking of oncogenes (e.g., *MEIS1*, *MYC*) refine emerging biological AML subgroups and highlight vulnerabilities to cohesin or bromodomain and extra terminal domain inhibition [70,73]. Hi-C also facilitates the study of epigenomic changes during AML differentiation and treatment response. With advances in single-cell and low-input Hi-C, it can be applied to rare cell populations or primary patient samples [74]. Current limitations—namely, the lack of SNV detection, the need for specialized bioinformatic pipelines, and higher sequencing-depth-related costs (diagnostic grade Hi-C ~35 kilobase resolution costs ≈ USD 1800–2000 per sample)—still confine Hi-C to selected cases, particularly those with ambiguous cytogenetics or suspected complex genomes. Also, the VAF of 10–20% is a clear limit for monitoring MRD [69,71,75]. Ongoing integration with long-read WGS [76] and OGM [77] is expected to close these gaps. It may support the incorporation of Hi-C-derived metrics into future WHO and ELN classification frameworks.

## 9. Machine Learning and Artificial Intelligence

Machine learning (ML) and artificial intelligence (AI) are increasingly applied across multiple aspects of AML care, improving accuracy, efficiency, and predictive power in diagnosis, risk stratification, treatment planning, and monitoring [78]. There are various examples of the successful implementation of different algorithms. AI and machine ML have shown significant potential in the management of AML. For instance, AI models can be used to classify peripheral blood images to detect cells characteristic of AML. This method was validated using 42,386 single-cell images from peripheral blood slides of 282 patients, including 82 diagnosed with AML. The deep learning model was specifically trained to differentiate between AML and acute lymphoblastic leukemia by accurately classifying myeloblasts and lymphoblasts [79]. In another study, an ML was developed to analyze RNA sequencing data and identify genes associated with programmed cell death. This led to the establishment of the pan-programmed cell death-related genes index. A higher score was correlated with a worse prognosis, suggesting that it could serve as a valuable prognostic biomarker and a screening tool for risk assessment in AML [80].

Additionally, ML has been applied to support clinical decision-making regarding the suitability of patients for hematopoietic stem cell transplantation. An ensemble model combining ML algorithms with clinical guidelines has been developed to improve AML risk stratification and provide recommendations on transplantation eligibility [78].

AI also holds promise for establishing personalized treatment strategies. Computational methods and ML techniques have been used to identify differential high-risk genes in AML, allowing for the classification of patients into low- and high-risk groups. Patients in the low-risk group demonstrated significantly longer overall survival. Moreover, these models can assist in recommending more tailored drug treatments based on individual genetic profiles [81].

## 10. Summary and Future Directions

We summarize the advantages, limitations, and other characteristics of the mentioned methods in the table below (Table 3).

The landscape of genetic assessment in AML is rapidly evolving, moving towards a more integrated and technologically advanced future. Current diagnostic standards, which rely on at least two genetic methods—typically CCA, and a molecular genetic technique like NGS panels—are effective, but have limitations [6,16,17]. The future of AML diagnostics and management will likely focus on overcoming these analytical gaps through the adoption of more comprehensive and sensitive technologies, fully leveraging the power of AI, and aiming for truly personalized medicine

A primary future direction is the shift towards a single, all-encompassing genetic test. WGS stands out as a strong candidate to replace the current multi-test approach, as it can detect all classes of genetic alterations, from SNVs to large structural changes, in one assay. While cost and complex data interpretation are current barriers, these are expected to diminish, potentially establishing WGS as the new standard of care [49,50,51].

Similarly, other novel methods show great promise for routine clinical use. OGM offers a high-resolution view of structural variants, potentially replacing CCA as the gold standard for cytogenetic analysis. Its combination with targeted NGS has already been shown to significantly increase the detection of clinically relevant aberrations [59,60]. Long-read sequencing is another powerful tool, adept at identifying large and complex structural variants that are often missed by short-read technologies (Bravo-Perez, Au, and Espinoza). Hi-C analysis will also play a crucial role, particularly in cases with ambiguous cytogenetics, by uncovering cryptic rearrangements and providing insight into the three-dimensional organization of the genome. As the costs of these technologies decrease, using them in combination will allow for a multi-layered and highly detailed understanding of each patient’s unique AML subtype [59,60,64,65,66,69,70,71].

The integration of AI and ML will undoubtedly be indispensable in this new era. These technologies are already demonstrating their ability to enhance diagnostic accuracy, refine risk stratification, and guide treatment decisions [79,80,81]. In the future, AI algorithms will be essential for interpreting the massive datasets generated by WGS, OGM, and Hi-C, identifying novel prognostic biomarkers, and predicting patient response to specific therapies. ML models will continue to be refined for applications such as analyzing peripheral blood images, supporting hematopoietic stem-cell transplantation decisions, and developing personalized drug treatment strategies based on individual genetic profiles

Ultimately, these advancements will converge to deliver on the promise of personalized medicine. A more profound and comprehensive genetic diagnosis will enable more accurate risk stratification and the selection of targeted therapies. The ability to monitor MRD with greater sensitivity using advanced sequencing techniques will allow for timely intervention and adjustment of treatment plans, improving overall patient outcomes. The ongoing evolution of diagnostic technologies, coupled with the power of computational analysis, heralds a future where every AML patient receives a tailored treatment strategy based on a complete and nuanced understanding of their disease’s genetic underpinnings.

## 11. Conclusions

Current guidelines for diagnosing and managing AML patients use at least two genetic methods, usually CCA and a molecular genetic technique, for accurate genetic diagnosis, risk assessment, and prognosis. The need for quick, precise information about the current genetic background highlights the importance of this additional molecular data. As a standard practice, NGS panels remain reliable, and are continuously expanding in scope. However, other methods such as Hi-C, OGM, and broader sequencing techniques could also be helpful, and show great potential for future diagnostic and treatment strategies. As costs are expected to decrease over time, combining two or more methods could help fill known analytical gaps. Moreover, like in many areas today, AI and ML are increasingly becoming essential partners in data management and improving patient care. Therefore, adopting new approaches would increase the likelihood of personalized treatment and better outcomes for patients.

## Figures and Tables

**Table 1:** WHO 2022 classification * (adapted by Khoury et al., 2022 [3]).

**Acute myeloid leukaemia with defining genetic abnormalities**
Acute promyelocytic leukaemia with *PML*::*RARA* fusion
Acute myeloid leukaemia with *RUNX1*::*RUNX1T1* fusion
Acute myeloid leukaemia with *CBFB*::*MYH11* fusion
Acute myeloid leukaemia with *DEK*::*NUP214* fusion
Acute myeloid leukaemia with *RBM15*::*MRTFA* fusion
Acute myeloid leukaemia with *BCR*::*ABL1* fusion
Acute myeloid leukaemia with *KMT2A* rearrangement
Acute myeloid leukaemia with *MECOM* rearrangement
Acute myeloid leukaemia with *NUP98* rearrangement
Acute myeloid leukaemia with *NPM1* mutation
Acute myeloid leukaemia with *CEBPA* mutation
Acute myeloid leukaemia, myelodysplasia-related
Acute myeloid leukaemia with other defined genetic alterations
**Acute myeloid leukaemia, defined by differentiation**
Acute myeloid leukaemia with minimal differentiation
Acute myeloid leukaemia without maturation
Acute myeloid leukaemia with maturation
Acute basophilic leukaemia
Acute myelomonocytic leukaemia
Acute monocytic leukaemia
Acute erythroid leukaemia
Acute megakaryoblastic leukaemia

* Additional footnotes can be seen in the original reference.

**Table 2:** ELN 2022 Risk stratification * (Döhner et al., 2022 [6]).

Risk Category	Genetic Abnormality
Favorable	t(8;21)(q22;q22.1)/*RUNX1*::*RUNX1T1* inv(16)(p13.1q22) or t(16;16)(p13.1;q22)/*CBFB*::*MYH11* Mutated *NPM1* without *FLT3*-ITD bZIP in-frame mutated *CEBPA*
Intermediate	Mutated *NPM1* with *FLT3*-ITD Wild-type *NPM1* with *FLT3*-ITD (without adverse-risk genetic lesions) t(9;11)(p21.3;q23.3)/*MLLT3*::*KMT2A* Cytogenetic and/or molecular abnormalities not classified as favorable or adverse
Adverse	t(6;9)(p23.3;q34.1)/*DEK*::*NUP214* t(v;11q23.3)/*KMT2A*-rearranged t(9;22)(q34.1;q11.2)/*BCR*::*ABL1* t(8;16)(p11.2;p13.3)/*KAT6A*::*CREBBP* inv(3)(q21.3q26.2) or t(3;3)(q21.3;q26.2)/*GATA2*, *MECOM(EVI1)* t(3q26.2;v)/*MECOM*(*EVI1*)-rearranged −5 or del(5q); −7; −17/abn(17p) Complex karyotype, monosomal karyotype Mutated *ASXL1*, *BCOR*, *EZH2*, *RUNX1*, *SF3B1*, *SRSF2*, *STAG2*, *U2AF1*, and/or *ZRSR2* Mutated *TP53*

* Additional footnotes can be seen in the original reference.

**Table 3:** Characteristics of the methods for genomic valuation of AML.

Method	Advantages	Limitations	Sensitivity and Resolution	TAT	MRD Detection	Cost per Sample	Routine Use
**CCA**	Whole-genome assessment of large-scale alterations. Essential for diagnosis, as its markers are included in WHO 2022 classification and ELN 2022 risk stratification.	Poor resolution. Can miss clones with less than 10% sensitivity.	10% for clones 5–10 megabases	7–21 days	No	USD 150	Yes, gold-standard
**NGS gene panels**	Simultaneous evaluation of multiple genes Detects single-nucleotide variations, CNAs, translocations, and indels. Contributes to therapy selection and relapse risk assessment.	Relatively high price.	Up to 0.01% SNVs	2–7 days for rapid results	Yes	A few hundred–a few thousand USD	Yes, standard of care
**WES**	Useful as a diagnostic tool when targeted gene panels are inconclusive.	Limited in detecting large SVs and CNAs. Blind to 99% of the genome.	5–10% VAF SNVs	A few weeks	No	Up to USD 2000	No
**WGS**	Detects all classes of genetic alterations in a single assay. Shows high concordance with established methods, while uncovering additional clinically relevant information.	High costs and massive data storage needs vast numbers of VUSs.	5–10% VAF SNVs	Longer than WES, rapid results possible in a few days.	No	Up to USD 4000	No
**SNP-microarray**	Detects microdeletions, amplifications, and loss of heterozygosity, and interstitial deletions in about 25% of common fusion genes. Clarifies marker and ring chromosomes.	Cannot report balanced changes. May miss clones with less than 10% VAF. Cannot detect minor single-gene variants.	10% VAF 5–10 kilobases	14–21 days	No	A few hundred USD	Yes, but not as a sole molecular-genetic method
**OGM**	Identifies CNAs and SVs, potentially doubling the number of findings compared to CCA. Could establish itself as a new gold standard for cytogenetic changes.	Higher cost than CCA. Reduced sensitivity for Robertsonian translocations, compared to CCA.	5% VAF 500 bp resolution	7–14 days	No	USD 500	Yes
**Long-read sequencing**	Detects large SVs often missed by short-read sequencing. Has been used to successfully detect novel and cryptic translocations in AML.	Historically higher raw read error rates, Higher per-sample costs, lower throughput, and need for specialized bioinformatics.	≥10% VAF SNVs	3–14 days, depending on the platform.	No	Highly variable. depending on the platform; on average, a few thousand USD.	No
**Hi-C analysis**	Genome-wide detection of large-scale rearrangements and gene fusions missed by other methods. Uncovers additional clinically actionable fusions in cases with cryptic karyotypes.	Does not detect single-nucleotide variants. Requires specialized bioinformatic pipelines. Higher costs, related to sequencing depth.	≥10–20% VAF 35 kilobases	Not standardized for clinical use; the lab process is multi-day, and complex data analysis adds significant time.	No	USD 1800–2000	No

## Data Availability

This study did not create or analyze new data, and data sharing does not apply to this article.

## Abbreviations

The following abbreviations are used in this manuscript:

AI	artificial intelligence
AML	acute myeloid leukemia
Bp	base pairs
CCA	conventional cytogenetic analysis
CNAs	copy number alterations
ELN	European Leukemia Net
Hi-C	High-throughput chromosome conformation capture
ICC	International Consensus Classification
MDS	myelodysplastic syndrome
MRD	measurable residual disease
NGS	next-generation sequencing
OGM	optical genome mapping
SNP	single-nucleotide polymorphism
SNV	single-nucleotide variant
SV	structural variant
TADs	topologically associating domains
TAT	turnaround time
VAF	variant allele frequency
VUS	variant of unknown significance
WES	whole-exome sequencing
WGS	whole-genome sequencing
WHO	World Health Organization
